# Sarcopenia among people living with HIV and the effect of antiretroviral therapy on body composition

**DOI:** 10.1097/MD.0000000000031349

**Published:** 2022-10-21

**Authors:** Keiji Konishi, Hidenori Nakagawa, Tomohiro Asaoka, Yu Kasamatsu, Tetsushi Goto, Michinori Shirano

**Affiliations:** a Department of Infectious Diseases, Osaka City General Hospital, Osaka, Japan; b Department of Infectious Diseases, University Hospital Kyoto Prefectural University of Medicine, Kyoto, Japan; c Department of Infectious Diseases, Osaka City Juso Hospital, Osaka, Japan.

**Keywords:** antiretroviral therapy, body composition, Japan, people living with HIV, sarcopenia, weight gain

## Abstract

To investigate the prevalence of sarcopenia among people living with HIV (PLWH) in Japan and analyze the relationship between HIV infection and ART effects on the body composition of Japanese PLWH for more appropriate drug selection and lifestyle guidance. Cross-sectional observational study. We included male patients aged ≥ 60 years whose body composition was measured by InBody 570 body composition analyzer during outpatient visits. Patients were classified by body shape based on body mass index (BMI) and body fat percentage measurements and by tenofovir alafenamide administration. Hidden obesity is a condition wherein the BMI is within the standard range but the body fat percentage is higher than the reference. Patients with low muscle mass and strength were considered to have sarcopenia, whereas those with only low muscle strength were considered to have pre-sarcopenia. In total, 87 patients were included. Based on body shape determined by BMI and body fat percentage, most patients had hidden obesity (40 patients, 46.0%). Sarcopenia was detected in 9 patients (10.3%) and pre-sarcopenia in 14 patients (16.1%). The tenofovir alafenamide (TAF) use group had significantly higher BMI, higher skeletal muscle mass, body fat mass, and skeletal muscle mass index relative to the non-TAF use group. Hidden obesity is a risk for lifestyle diseases. It is important to recognize it based on body composition measurements because it can be missed by BMI measurement alone. Tenofovir alafenamide therapy increases skeletal muscle mass, which may result in the prevention of sarcopenia. To clarify how TAF affects the development of sarcopenia and lifestyle diseases, future studies on a larger cohort are warranted.

## 1. Introduction

After the introduction of antiretroviral therapy (ART) (a combination of three or more anti-HIV drugs), the life expectancy of people living with HIV (PLWH) improved significantly. However, concerns about long-term complications of ART, such as hypertension, diabetes, dyslipidemia, and weight gain have recently arisen.^[1]^ Hidden obesity is a condition wherein the body mass index (BMI) is within the standard range but the body fat percentage is higher than the reference, and although hidden obesity may be overlooked by weight measurements alone, the risk of lifestyle diseases in patients with hidden obesity is notably as high as that in obese patients. The aging of PLWH is also a problem in Japan, and the age distribution of HIV infection/AIDS reported between 2015 and 2020 shows the largest proportion in the 20 to 40 years group, which is considered sexually active but at least 6% of PLWH are over 60 years old. Furthermore, more than half of those over 60 years who developed AIDS are HIV-infected.

Although the management of long-term complications has become important in extending the healthy life span of PLWH, sarcopenia has recently been studied not only among PLWH but also among non-PLWH. Characterized by low muscle mass and low muscle function (muscle strength or physical function) with aging, sarcopenia is a progressive and systemic skeletal muscle disease with an increased risk of health problems. It is associated with clinical outcomes, such as falls, frailty, osteoporosis, fractures, dysphagia, and low nutrition, which have been reported to be associated with lifestyle diseases and chronic inflammatory diseases, including HIV infection.^[2,3]^ Sarcopenia can be broadly classified into primary sarcopenia caused by aging and secondary sarcopenia caused due to long periods of inactivity, such as being bedridden and having inflammatory diseases, including HIV infection, lifestyle diseases, and malnutrition due to malabsorption.^[4]^ There are three different stages of sarcopenia as follows: pre-sarcopenia (low muscle mass), sarcopenia (low muscle mass and low muscle strength or decreased physical function), and severe sarcopenia (low muscle mass, low muscle strength, and decreased physical function).^[4]^ Pre-sarcopenia is a preliminary stage of sarcopenia that is considered to have the potential of leading to sarcopenia.

With aging among PLWH, the number of PLWH who develop frailty at an early age is increasing.^[2]^ PLWH has a significant decrease in skeletal muscle mass, walking speed, and grip strength compared with uninfected patients.^[3]^ Drugs used for ART are speculated to cause mitochondrial dysfunction and muscle damage; however, the causal relationship between sarcopenia and current ART drugs with reduced toxicity has not yet been investigated.^[4]^

In Japan, the prevalence of sarcopenia in healthy individuals aged ≥ 65 years is 7.5%,^[5]^ whereas that among PLWH outside of Japan is 24.1%,^[6]^ which is 6.1 times higher than the risk in non-PLWH. Multiple diagnostic criteria for sarcopenia are available, including those by the European Working Group on Sarcopenia in Older People^[2]^ and the Asian Working Group for Sarcopenia (AWGS)^[7]^; however, there is no global consensus on which criteria to use. In Japan, guidelines for sarcopenia^[8]^ were only established in 2017, and there are no reports on complications of sarcopenia in PLWH. Therefore, this study aimed to investigate the prevalence of sarcopenia based on the AWGS diagnostic criteria^[7]^ among PLWH attending outpatient clinics, the clinical characteristics of these patients, and the effect of ART on body composition.

## 2. Methods

### 2.1. Study design and population

We conducted a cross-sectional observational study at the Department of Infectious Diseases, Osaka City General Hospital (Osaka, Japan), an AIDS core base hospital in Japan with more than 700 PLWH visiting regularly. This study included HIV-infected male patients who visited the hospital’s outpatient clinic between April 1, 2021, and September 30, 2021. Women were excluded from the study because their body composition, including body fat percentage, differed significantly from that of men. The inclusion criteria were age ≥ 60 years and availability of measured data on body composition and grip strength. Of the 700 outpatients, 14% were aged ≥ 60 years. Patients who were <12 weeks post ART change at the time of InBody570 measurement, those whose body composition could not be measured due to difficulty in standing, and those who did not give consent to participate were excluded.

The study was approved by the Institutional Review Board of the Osaka City General Hospital as a single-center study and was conducted in compliance with the Declaration of Helsinki (approval number 2105018).

### 2.2. Measurements

Body composition measurement using the InBody 570 body composition analyzer was added to the items evaluated during a regular medical checkup. The survey items comprised body composition, patient information, blood and biochemical test values, and muscle strength.

#### 2.2.1. Diagnosis of sarcopenia

Sarcopenia was diagnosed according to the diagnostic criteria of the AWGS,^[7]^ i.e., grip strength (<28 kg) and muscle mass loss [skeletal muscle mass index (SMI): skeletal muscle mass of the extremities divided by the square of height] < 7.0 kg/m^2^. Gait speed was not used because of the difficulty in securing a measurement site and because it is not a required item in the revised European Working Group on Sarcopenia in Older People 2 criteria,^[9]^ which used gait speed to determine the disease severity. Patients with SMI < 7.0 kg/m^2^ were classified in the pre-sarcopenia group, and those with ≥ SMI 7.0 kg/m^2^ and grip strength ≥ 28 kg were classified in the non-sarcopenia group.

#### 2.2.2. Body composition

Body composition was measured using a body composition analyzer (InBody 570 body composition analyzer, InBody Japan Co. Ltd., Tokyo, Japan) based on the bioelectrical impedance analysis method. The following items were measured: height, weight, BMI, body fat mass, lean body mass, body fat percentage, SMI, and visceral fat level (truncated one-digit value of visceral fat cross-sectional area estimated from impedance) were extracted from the measurement results.

BMI was classified according to the obesity criteria of the Japan Society for the Study of Obesity: underweight (<18.5 kg/m^2^), normal (18.5–25 kg/m^2^), and obese (>25 kg/m^2^). Regarding body fat mass, a body fat percentage of 10% to 20% was defined as the normal range, and that of ≥20% as high. Based on the BMI and body fat percentage data, patients were classified into the following groups according to body type: obese group: BMI ≥ 25 kg/m^2^ and body fat percentage ≥ 20%; hidden obese group: BMI ≥ 18.5 kg/m^2^ or < 25 kg/m^2^ and body fat percentage ≥ 20%; obese group: BMI ≥ 25 kg/m^2^ and body fat percentage < 20%; normal group: BMI 18.5–25 kg/m^2^ and body fat percentage < 20%; and thin group: BMI < 18.5 kg/m^2^.

#### 2.2.3. Patient information

The following clinical data were extracted from eligible patients: age, medical history of AIDS, lowest CD4-positive T lymphocyte count in the lifetime (nadir CD4), duration of ART, drugs of ART at the time of InBody measurement, and current use of medications for hypertension, diabetes, and dyslipidemia.

#### 2.2.4. Blood analysis

The following blood test measurements were included: CD4-positive T lymphocyte count, CD8-positive T lymphocyte count, HIV-RNA level, aspartate aminotransferase (AST), alanine aminotransferase (ALT), gamma-glutamyl transferase (γGTP), total protein, albumin, creatinine, uric acid, high-density lipoprotein cholesterol, low-density lipoprotein cholesterol, triglyceride (TG), HbA1c level, and C reactive protein level.

#### 2.2.5. Muscle strength

Muscle strength was evaluated by measuring the grip strength twice on each side, and the maximum value was recorded. Grip strength was measured using a Smedley-type grip strength meter (M-type grip strength meter, Matsumiya Medical Precision Instruments Co., Fukui, Japan).

### 2.3. Statistical analysis

Body shapes were classified into five categories based on BMI and body fat percentage. Comparisons of body composition were made between the normal and hidden obesity groups. Comparisons of body composition were made among the three groups, including non-sarcopenia, pre-sarcopenia, and sarcopenia, classified by SMI and grip strength. To examine the effects of ART on body composition, patients were also divided by ART. The patients were divided into two groups, the tenofovir alafenamide (TAF)-using and non-using as the backbone, and the integrase strand transfer inhibitor (INSTI), protease inhibitor (PI), and NNRTI groups by key drugs. The body composition was compared among groups. For continuous variables, the Mann–Whitney *U* test was used to compare differences between two groups, and the Kruskal–Wallis test among three groups. For categorical variables, including participant characteristics, the chi-square test or Fisher’s exact test was used for comparison. Statistical significance was set at a two-sided *P* < .05. All statistical analyses were performed using IBM SPSS, version 28.0 (IBM, Armonk, NY). Clinical data expressed as continuous variables are described as the median and interquartile range [first quartile–third quartile].

## 3. Results

In total, 87 patients were included in this study. The background of the patients at the time of body composition measurement is shown in Table 1. The median age was 68 [63–72] years. Based on body types classified according to BMI, 5 patients were underweight, 55 standard, and 27 obese. The most common ART used was bictegravir/tenofovir alafenamide/emtricitabine (n = 20), followed by dolutegravir (DTG) + abacavir/lamivudine (n = 12), DTG + TAF/FTC (n = 12), raltegravir + TAF/FTC (n = 12). As the backbone drug, TAF/FTC was used in 57 patients, abacavir/lamivudine in 17, two-drug therapy in 11, and tenofovir disoproxil fumarate/FTC in 2. As the key drug, 76 patients were treated with INSTI, 7 with non-nucleoside reverse transcriptase inhibitor (NNRTI), and 4 with PI.

**Table 1:** Characteristics and measurements for PLWH aged ≧ 60 years included in this study (N = 87).

Variables	Value
Age (yr)	68 [63–72]
Height (cm)	165 [163–172]
Body weight (kg)	65.6 [58.1–71.6]
BMI (kg/m^2^)	23.2 [20.8–25.5]
Development of AIDS	40 (46.0)
Nadir CD4 (/µL)	93 [24–183]
Duration since HIV diagnosis (yr)	11 [7–15]
Duration of ART (mo)	138 [90–183]
Use of TAF	57 (65.5)
Key drug	
INSTI	76
NNRTI	7
PI	4
Hypertension treatment	32 (36.8)
Diabetes treatment	14 (16.1)
Lipid disorder treatment	13 (14.9)
Body fat mass (kg)	16.6 [12.5–21.4]
Lean body mass (kg)	48.4 [44.6–52.0]
Body fat percentage (%)	24.7 [20.8–30.3]
Grip strength (kg)	35 [28–40]
Visceral fat level	7 [5–9]
SMI (kg/m^2^)	7.3 [6.9–7.9]

Data are presented as counts (percentages) or median [interquartile range].

ART = antiretroviral therapy, BMI = body mass index, INSTI = integrase strand transfer inhibitor, NNRTI = non-nucleoside reverse transcriptase inhibitor, PI = protease inhibitor, PLWH = people living with HIV, SMI = skeletal muscle mass index, TAF = tenofovir alafenamide.

### 3.1. Body composition measurement

**Figure 1.**
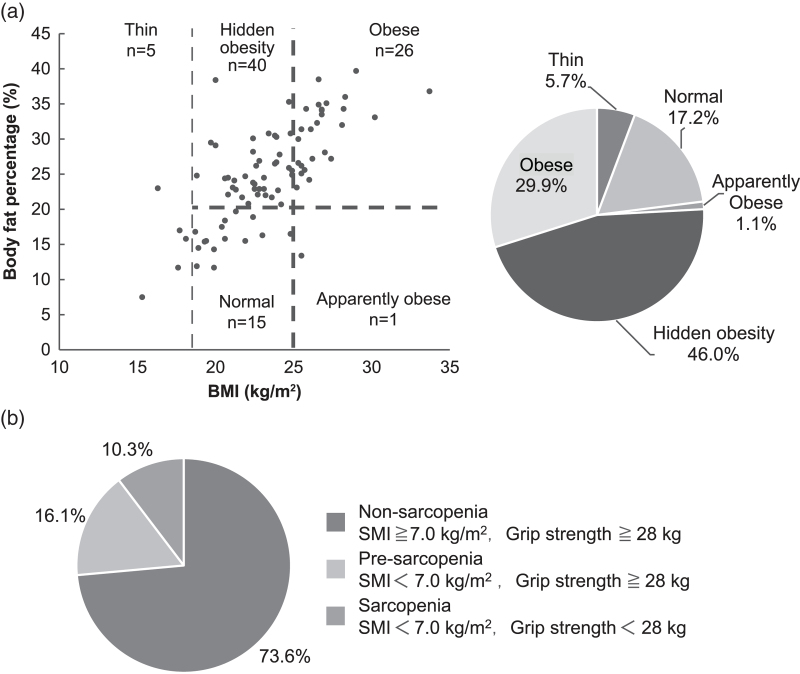
Body composition measurements for HIV-infected patients and prevalence of sarcopenia. (A) Body composition measurements for HIV-infected patients. (B) Prevalence of sarcopenia. BMI = body mass index, SMI = skeletal muscle mass index.

The distribution of patients based on body shape (assessed by BMI and body fat percentage measurements) is shown in Figure 1A. There were 27 patients (29.9%) in the obese group, 40 (46.0%) hidden obese, 1 (1.1%) obese, 15 (17.2%) normal, and 5 (5.7%) thin. Based on body fat percentage, the 55 patients classified as normal by BMI were divided into the normal (n = 15) and hidden obesity group (n = 40). A comparison of body composition between the two groups is shown in Table 2. Body weight, BMI, body fat mass, body fat percentage, and visceral fat level were significantly higher in the hidden obesity group than in the normal group. However, there was no significant difference in lean body mass, grip strength, or SMI.

**Table 2:** Characteristics and anthropometric values of the BMI standard group stratified by body fat percentage.

	Normal (n = 15)	Hidden obesity (n = 40)	*P*
Age (yr)	64 [61–72]	66 [61–72]	.887
Body weight (kg)	57.4 [52–62.4]	64.3 [59.2–69.2]	.002
BMI (kg/m^2^)	20.4 [19.3–21.9]	22.8 [21.4–23.9]	<.001
Development of AIDS	7 (46.7)	15 (37.5)	.537
Nadir CD4 (/µL)	144 [60–160]	96 [24–203]	.533
Duration since HIV diagnosis (yr)	13 [10–17]	10 [3–14]	.140
Duration of ART (mo)	148 [121–184]	123 [46–159]	.117
Use of TAF	7 (46.7)	27 (67.5)	.157
Key drug			
INSTI	13	36	
NNRTI	1	2	
PI	1	2	
Hypertension treatment	5 (33.3)	16 (40.0)	.650
Diabetes treatment	2 (13.3)	7 (17.5)	.710
Lipid disorder treatment	0	8 (20.0)	.061
Body fat mass (kg)	8.8 [8.0–12.1]	16.1 [13.5–17.7]	<.001
Lean body mass (kg)	47.2 [44.4–50.7]	48.2 [45.0–51.0]	.719
Body fat percentage (%)	15.8 [14.5–17.5]	24.6-22.8–28.1	<.001
Grip strength (kg)	37 [33–42]	35.5 [28.0–40.0]	.185
Visceral fat level	4 [3–5]	7 [6–7]	<.001
SMI (kg/m^2^)	7.2 [6.8–7.4]	7.3 [6.9–7.8]	.218

Data are presented as counts (percentages) or median [interquartile range].

Standard group < 20; hidden obesity group ≥ 20.

AIDS = acquired immunodeficiency syndrome, ART = antiretroviral therapy, BMI = body mass index, INSTI = integrase strand transfer inhibitor, NNRTI = non-nucleoside reverse transcriptase inhibitor, PI = protease inhibitor, SMI = skeletal muscle mass index, TAF = tenofovir alafenamide.

### 3.2. Prevalence and clinical features of sarcopenia

There were 9 (10.3%) patients in the sarcopenia group, 14 (16.1%) in the pre-sarcopenia group, and 64 (73.6%) in the non-sarcopenia group (Fig. 1B). According to body shape, the sarcopenia group (n = 9) had 5 hidden obese patients. In the pre-sarcopenia group (n = 14), 6 patients were classified as hidden obese.

The background and physical measurements of patients stratified by sarcopenia are shown in Table 3. Significant differences in weight, BMI, TAF use, body fat mass, lean body mass, grip strength, visceral fat level, and SMI were found among the three groups. The pre-sarcopenia and sarcopenia groups tended to have lower body weight, BMI, body fat mass, and lean body mass than the non-sarcopenia group. The sarcopenia groups also tended to have a lower TAF use than the non-sarcopenia and pre-sarcopenia groups.

**Table 3:** Background and physical measurements for PLWH stratified by sarcopenia stage (N = 87).

	Non-sarcopenia (n = 64)	Pre-sarcopenia (n = 14)	Sarcopenia (n = 9)	*P*
Age (yr)	66 [62–72]	67.5 [63–72]	71 [64–75]	.527
Body weight (kg)	67.7 [63.2–73.4]	57.1 [48.4–60.3]	56.1 [53.2–61.9]	<.001
BMI (kg/m^2^)	24.1 [22.5–26.1]	20.4 [18.0–22.2]	20.6 [19.5–23.4]	<.001
Development of AIDS	29 (45.3)	6 (42.9)	5 (55.6)	.819
Nadir CD4 (/µL)	73 [21–150]	170 [50–226]	74 [36–289]	.128
Duration since HIV diagnosis (yr)	11 [7–15]	11 [4–13]	14 [13–15]	.483
Duration of ART(mo)	132 [90–184]	135 [38–182]	157 [95–178]	.682
Use of TAF	46 (71.9)	9 (64.3)	2 (22.9)	.013
Key drug				
INSTI	54	13	9	
NNRTI	6	1	0	
PI	4	0	0	
Hypertension treatment	27 (42.2)	3 (21.4)	2 (22.2)	.218
Diabetes treatment	9 (14.1)	4 (28.6)	1 (11.1)	.372
Lipid disorder treatment	11 (17.2)	1 (7.1)	1 (11.1)	.598
Body fat mass (kg)	17.6 [13.5–22.1]	11.4 [7.8–14.7]	13.7 [9.1–20.4]	.002
Lean body mass (kg)	50.1 [47.2–53.5]	43.5 [41.5–46.5]	44.0 [38.7–45.7]	<.001
Body fat percentage (%)	25.8 [22.0–30.8]	19.9 [15.5–24.6]	24.4 [17.7–32.4]	.055
Grip strength (kg)	36.5 [30–40.8]	33 [30.3–36]	24 [19–26]	<.001
Visceral fat level	7 [6–10]	5 [3–6]	6 [4–10]	.002
SMI (kg/m^2^)	7.6 [7.3–7.9]	6.6 [6.3–6.8]	6.6 [5.4–6.9]	<.001
CD4^+^ cell count (/µL)	500 [364–586]	448 [339–603]	562 [508–703]	.227
CD8^+^ cell count (/µL)	713 [557–1015]	848 [551–955]	795 [488–856]	.857
HIV-RNA level (copies/mL)	20 [20–20]	20 [20–30]	20 [20–20]	.524
CD4/CD8	0.69 [0.46–0.91]	0.56 [0.40–0.86]	0.89 [0.51–1.36]	.285
AST (U/L)	23 [19–27]	19 [16–24]	22 [19–25]	.124
ALT (U/L)	18 [15–25]	13 [12–17]	18 [15–19]	.011
γGTP (U/L)	27 [18–29]	24 [15–34]	16 [15–27]	.061
Total protein (g/dL)	7.4 [7.0–7.6]	7.1 [6.8–87.5]	7.2 [7.1–7.5]	.271
Albumin (g/dL)	4.2 [4.0–4.4]	4.1 [3.9–4.4]	4.1 [3.9–4.5]	.728
Creatinine (mg/dL)	0.96 [0.86–1.09]	0.85 [0.74–1.16]	0.84 [0.74–0.96]	.046
Uric acid (mg/dL)	5.8 [5.0–6.7]	5.2 [4.6–6.3]	6.3 [5.2–6.4]	.304
HDL-C (mg/dL)	50 [43–56]	51 [44–63]	51 [42–62]	.895
LDL-C (mg/dL)	110 [91–131]	95 [82–117]	101 [98–125]	.211
TG (mg/dL)	152 [131–229]	128 [74–170]	125 [62–146]	.031
HbA1c (%)	5.7 [5.4–6.1]	5.7 [5.4–7.1]	5.8 [5.5–6.4]	.867
CRP (mg/dL)	0.07 [0.03–0.18]	0.05 [0.03–0.24]	0.03 [0.03–0.19]	.258

Data are presented as counts (percentages) or median [interquartile range].

ALT = alanine aminotransferase, ART = antiretroviral therapy, AST = aspartate aminotransferase, BMI = body mass index, CRP = C reactive protein, HDL-C = high density lipoprotein cholesterol, INSTI = integrase strand transfer inhibitor, LDL-C = low density lipoprotein cholesterol, NNRTI = non-nucleoside reverse transcriptase inhibitor, PI = protease inhibitor, PLWH = people living with HIV, SMI = skeletal muscle mass index, TAF = tenofovir alafenamide, TG = triglyceride.

Regarding blood test measurements, significant differences in ALT, creatinine, and TG levels were noted; ALT levels tended to be lower in the pre-sarcopenia group than in the other two groups, and creatinine and TG levels tended to be higher in the non-sarcopenia group.

### 3.3. Background and physical measurements classified by ART

The background and physical measurements classified by ART are shown in Table 4. Weight, BMI, duration of ART, body fat mass, lean body mass, grip strength, and SMI were significantly higher in the TAF group (n = 57) than in the non-TAF group (n = 30). A significantly higher lean body mass was noted in the PI group (n = 4) than in the INSTI (n = 76) and NNRTI (n = 7) groups.

**Table 4:** Background and physical measurements of patients stratified by ART drugs.

	TAF group (n = 57)	Non-TAF group (n = 30)	*P*	INSTI (n = 76)	NNRTI (n = 7)	PI (n = 4)	*P*
Age (yr)	66 [62–71]	71 [64–73]	.022	68 [63–72]	70 [62–73]	63 [61–68]	.361
Body weight (kg)	67.3 [61.9–73.6]	58.9 [53.4–67.5]	<.001	65.0 [57.5–70.9]	72.8 [59.4–82.2]	52.6 [66.0–77.4]	.146
BMI (kg/m^2^)	23.8 [22.0–25.7]	22.2 [19.6–24.8]	.020	23.1 [20.8–25.5]	23.2 [19.4–26.8]	23.7 [22.7–26.5]	.753
Development of AIDS	22 (28.6)	18 (60.0)	.057	42 (55.3)	3 (42.9)	2 (50.0)	.809
Nadir CD4 (/µL)	99 [24–186]	69 [18–181]	.755	115 [24–198]	46 [21–144]	34 [17–146]	.399
Duration of ART (mo)	117 [59–148]	165 [141–218]	<.001	138 [88–175]	121 [53–286]	142 [104–211]	.823
Hypertension treatment	22 (38.6)	10 (33.3)	.628	28 (36.8)	3 (42.9)	1 (25.0)	.839
Diabetes treatment	9 (15.8)	5 (16.7)	.916	10 (13.2)	3 (42.9)	1 (25.0)	.109
Lipid disorder treatment	11 (19.3)	2 (6.7)	.116	11 (14.5)	1 (14.3)	1 (25)	.846
Body fat mass (kg)	17.6 [13.6–22.0]	15.3 [8.5–18.0]	.024	16.6 [12.6–20.7]	16.0 [9.2–27.1]	17.5 [13.0–21.3]	.906
Lean body mass (kg)	50.1 [46.7–53.8]	45.3 [42.3–49.6]	<.001	48.1 [44.3–51.0]	50.2 [47.4–56.8]	55.1 [52.5–56.6]	.016
Body fat percentage (%)	25.1 [21.7–31.0]	24.4 [17.0–29.2]	.330	24.8 [21.0–30.3]	22.9 [15.5–34.1]	24.1 [19.6–27.0]	.887
Grip strength (kg)	35 [30–40.5]	32 [25.8–37.3]	.022	35.0 [28.0–40.0]	32 [27–42]	36.5 [32.8–46.3]	.570
Visceral fat level	7 [6–10]	7 [4–7]	.059	7 [5–9]	7 [4–13]	8 [5–10]	.895
SMI (kg/m^2^)	7.6 [7.1–7.9]	7.1 [6.8–7.5]	.005	7.3 [6.9–7.8]	7.3 [7.3–8.1]	7.7 [7.3–8.0]	.376

Data are presented as counts (percentages) or median [interquartile range].

AIDS = acquired immunodeficiency syndrome, ART = antiretroviral therapy, BMI = body mass index, INSTI = integrase strand transfer inhibitor, NNRTI = non-nucleoside reverse transcriptase inhibitor, PI = protease inhibitor, SMI = skeletal muscle mass index, TAF = tenofovir alafenamide.

## 4. Discussion

This is the first study to investigate the anthropometric measurements of and prevalence of sarcopenia among PLWH in Japan. The results showed that hidden obesity was the most common type of obesity (46%) based on body composition assessed by BMI and body fat percentage and that 72.7% of the patients in the normal BMI group had a high body fat percentage. The prevalence of sarcopenia was 10.3%, which was not significantly different from that of healthy Japanese participants aged ≥ 65 years.^[5]^

Weight gain in PLWH due to ART and increased insulin resistance due to chronic inflammation^[10]^ is an important issue because it increases the risk of developing lifestyle diseases. Regimens including INSTI, the first-line ART in many countries worldwide including Japan, cause greater weight gain relative to other regimens, including NNRTI and PI, and treatment with TAF for NRTI causes greater weight gain than regimens with tenofovir disoproxil fumarate.^[1]^ In this study, the proportion of obese PLWH was 29.9%, which was like that reported in the 2021 National Health and Nutrition Survey in Japan (33.0%).^[11]^ We found no significant difference in BMI by the key drug; however, the TAF group had a significantly higher BMI than the non-TAF group. Additionally, the TAF group had a significantly higher body fat mass, lean body mass, and SMI than the non-TAF group. These results suggest that TAF-induced weight gain is associated not only the body fat but also the skeletal muscle mass.

In this study, 46.0% of the total population had hidden obesity, and 72.7% of the patients in the standard BMI group had hidden obesity. Among 1048 Japanese male workers (mean age: 41.8 years), 35.4% have hidden obesity^[12]^; however, there are only a few reports on the prevalence of hidden obesity in the elderly aged ≥ 60 years. Hidden obesity is a condition in which the BMI is within the standard range but the body fat percentage is higher than the standard range. Abnormal laboratory values like those of obesity characterize hidden obesity.^[13]^ Yamada et al classified Japanese men and women who underwent physical checkups into body shape groups (hidden obesity, normal, and obese) based on their BMI and body fat percentage and determined the factors related to lifestyle diseases. They reported that the pulse wave velocity, which is an indicator of arterial stiffness, high-density lipoprotein cholesterol, low-density lipoprotein cholesterol, and adiponectin, was significantly increased in the hidden obese and obese groups than in the normal group and that there was no significant difference between the hidden obese and obese groups.^[13]^ These results suggest that the prevention of lifestyle diseases is as important for patients with hidden obesity as it is for obese patients; however, caution is needed because it may be overlooked if body fat percentage is not measured.

The ratio of muscle mass is relatively lower in the hidden obesity group because of the high body fat percentage. In this study, the hidden obesity group had a significantly higher body fat mass than the normal group, with no significant differences in lean body mass or SMI. This suggests that the hidden obesity group had a relatively low muscle mass. Future decreases in muscle mass due to aging may lead to sarcopenia.

Furthermore, it is important to note that hidden obesity may lead to “sarcopenia obesity,” which is considered to increase the risk of physical function decline and development of metabolic diseases more significantly than sarcopenia because of its large amount of fat.^[14]^ Among the 23 patients diagnosed with sarcopenia and pre-sarcopenia, 11 (47.8%) patients were hidden obese; however, whether hidden obesity is a risk factor for sarcopenia in PLWH remains unclear.

Although there are no reports on hidden obesity in PLWH, abnormalities in the distribution of body fat (increased visceral fat in the abdomen and decreased subcutaneous fat in the limbs and face), called lipoatrophy, have been reported in patients receiving ART for a long period,^[15,16]^ which may be related to the cause of hidden obesity in PLWH. Although a clear cause is not known, lipoatrophy is speculated to involve mitochondrial dysfunction owing to NRTIs, as a previous study has reported a decrease in the amount of mitochondrial DNA in adipocytes.^[17]^ The higher frequency of lipoatrophy in users of stavudine (currently not available in Japan) is consistent with this hypothesis; however, the association between lipoatrophy and TAF, which has been used more frequently in recent years, is unclear. Whether hidden obesity in PLWH is associated with a higher risk of transitioning to sarcopenia or sarcopenic obesity is a subject for future research.

In this study, the prevalence of sarcopenia was 10.3% and that of pre-sarcopenia was 16.1% among PLWH aged ≥ 60 years. The prevalence of sarcopenia was comparable to that in healthy people aged ≥ 65 years in Japan, which was 7.5% (8.2% in men and 6.8% in women)^[5]^; however, the prevalence of pre-sarcopenia we obtained was lower than the reported prevalence of 43.2%^[18]^ in healthy men aged ≥ 65 years. In a systematic review and meta-analysis of 13 studies on sarcopenia among PLWH (N = 2267), the prevalence of sarcopenia was 24.1%.^[6]^ The risk of sarcopenia was 6.1 times higher than that of HIV-free people matched for age, sex, BMI, and ethnicity. Longer exposure to certain anti-HIV drugs (e.g., stavudine and didanosine), higher tobacco and alcohol use, lower educational level and employment rates, and longer duration of HIV infection was also associated with sarcopenia.^[6]^ Additionally, PLWH has a significant decrease in skeletal muscle mass and strength compared with uninfected individuals.^[4]^ We reported a lower sarcopenia prevalence in Japanese PLWH than those outside Japan and a lower pre-sarcopenia prevalence in PLWH than in healthy men and attribute these differences to the following factors: different diagnostic criteria, different methods of measuring SMI (dual-energy X-ray absorptiometry is more common in Europe and the U.S.), and influence of ART. We found that the use of TAF in the non-sarcopenia group was 71.9%, which has higher than that in the sarcopenia group (22.9%). If the weight gain from TAF is due to an increase in both body fat mass and skeletal muscle mass, the use of TAF may result in the prevention of sarcopenia.

This study had some limitations. First, we did not assess gait speed. Physical function is generally assessed by gait speed; however, the relationship between gait speed and lower limb muscle strength has been reported as nonlinear in community-dwelling and hospitalized patients with a mean age of 75 years.^[19]^ Since gait speed is related to various factors, such as the nervous system, stride length, range of motion of joints, balance function, and lower limb muscles, gait speed should be considered to evaluate the degree of sarcopenia. In this study, it was difficult to measure gait speed due to the difficulty in securing a measurement site; therefore, we could not find any cases of severe sarcopenia. Second, this was a single-center study. Third, dietary intake was not assessed. Fourth, only the drugs of ART at the time of the InBody measurement were extracted, and the history of ART was not investigated. Future studies should conduct nutritional surveys to examine the effects of active nutritional intervention by dietitians and improvement in long-term outcomes.

## 5. Conclusion

We revealed that the prevalence of hidden obesity is high among PLWH. Although hidden obesity may be overlooked by weight measurement alone, the risk of lifestyle diseases in hidden obese patients is notably as high as that in obese patients. Measurement of body fat percentage based not only on body weight but also on data from body composition analyzers may lead to early detection of hidden obesity. In recent years, weight gain due to ART, especially INSTI and TAF, has become a worldwide problem. However, weight gain due to TAF increased not only the body fat mass but also the skeletal muscle mass, which may have prevented sarcopenia. To clarify how TAF affects the development of sarcopenia and lifestyle diseases, future studies on a larger cohort are warranted. Early detection and intervention are important for sarcopenia and lifestyle diseases to maintain healthy life expectancy among PLWH.

## Acknowledgments

The authors wish to thank all the clinical staff of the Osaka City General Hospital for their help in the completion of this study. K.K. contributed to the study design, conducted the literature review, wrote the manuscript, and was responsible for the final decision of submission. H.N. revised the manuscript and provided important intellectual content. All authors have read and approved the final manuscript.

We would like to thank Editage (www.editage.com) for English language editing.

## Author contributions

**Conceptualization:** Keiji Konishi.

**Investigation:** Keiji Konishi, Tomohiro Asaoka, Yu Kasamatsu, Tetsushi Goto, Michinori Shirano.

**Project administration:** Keiji Konishi.

**Supervision:** Hidenori Nakagawa, Michinori Shirano.

**Writing – original draft:** Keiji Konishi.
